# Interaction between perceptual and cognitive processing well acknowledged in perceptual expertise research

**DOI:** 10.3389/fnhum.2014.00308

**Published:** 2014-05-20

**Authors:** Alan C.-N. Wong, Yetta K. Wong

**Affiliations:** ^1^Perception and Experience Laboratory, Department of Psychology, The Chinese University of Hong KongHong Kong, China; ^2^Department of Applied Social Studies, City University of Hong KongHong Kong, China

**Keywords:** perceptual expertise, object recognition, modularity, visual training, review

To understand the neural correlates of expert object recognition, Harel et al. (2013) proposed the use of an existing theoretical framework (Mahon et al., 2007; Martin, 2007) that emphasizes the interaction between different parts of the visual pathway as well as between the visual and other cognitive systems. While we agree that focusing more on the role of these interactions in expertise acquisition is a fruitful research direction, we would like to clarify the position of perceptual expertise researchers^1^. In fact, perceptual expertise researchers never regard face-selective areas as the only neural substrates important for expert object recognition. Nor do they deny the role of interaction between the visual system and other cognitive systems. Instead, perceptual expertise researchers have been considering the interaction between perceptual and cognitive processing as an important component in understanding perceptual expertise for different objects. It is therefore unnecessary to create the debate between the so-called “perceptual view” and “interactive view” of expert object recognition, as the interaction between perceptual and cognitive processing has been well accommodated in perceptual expertise research. We elaborate on this idea through the following two points:

## Perceptual expertise researchers do emphasize the role of attentional and higher-level cognitive factors in expertise acquisition and expression

Harel et al. (2013) states that according to perceptual expertise researchers, “expert processing… is automatic and stimulus-driven, with little impact of attentional, task demands or other higher-level cognitive factors” (p.2). Unfortunately, this characterization of the views of perceptual expertise researchers is inaccurate. Palmeri and Gauthier (2004), for example, proposes the abandoning of the strict distinction between perceptual and cognitive processes in understanding expert object recognition. Bukach et al. (2006), another landmark paper detailing the perceptual expertise framework, affirms that there are different kinds of perceptual expertise for different objects, and to distinguish between them both physical and conceptual (e.g., functional knowledge) properties should be considered.

Research has shown the importance of task demands in the *development* as well as *expression* of expertise in object recognition. Task demand during training is always a major factor determining whether or what kind of expertise would be formed (e.g., Tanaka et al., 2005; Scott et al., 2006; Krigolson et al., 2009; Wong et al., 2009a,b, 2012). Task demand during testing also affects whether and how much expertise effects can be observed (Wong et al., 2009b, 2012, 2014). For example, in Wong et al. (2009b), two groups of observers learned to categorize the same set of artificial objects (ziggerins) in different ways, leading to different changes of neural selectivity patterns for the trained objects. Importantly, the neural changes were better observed when the testing task matched the training task. Even the FFA shows higher activity to different objects in tasks that requires more attention such as old/new recognition than passive viewing (Rhodes et al., 2004). Therefore, using Harel et al.'s (2013) terms (their footnote 2), both “task-specific learning effects” and “task dependence following expertise training” have been well identified among perceptual expertise researchers (see also Box 2 of Bukach et al., 2006).

Perceptual expertise researchers put a lot of emphasis on the engagement of non-visual factors and the involvement of visual and non-visual areas outside the FFA (James and Gauthier, 2003, 2004, 2006; James and Atwood, 2009; James and Cree, 2010; Wong and Gauthier, 2010a; Bilalić et al., 2011a, 2010, 2012; Behrmann and Plaut, 2013; Kersey and James, 2013). For example, Wong and Gauthier (2010a) found that expert perception of musical notes engages not only higher visual regions that are distinct from the face- or letter-selective regions, but also bilateral early retinotopic cortex, and a wide range of multimodal regions including auditory, audiovisual, somatosensory, motor, parietal, frontal, and various subcortical areas. Similarly, a distributed network of areas including the motor and inferior frontal cortices is also engaged selectively for visual judgments of letters (James and Gauthier, 2006). A wide range of brain regions in the occipital, temporal, and frontal regions has also been found to be more active when chess experts performed visual judgment of chess pieces on chessboards (Bilalić et al., 2010; JEPG).

Training studies also show clearly the engagement of a widespread neural network of areas for expert object processing. When comparing the neural training effects of two traditions of visual perceptual training protocols (namely perceptual learning and perceptual expertise training), a wide range of brain regions has been investigated, including the recruitment and disengagement of early retinotopic cortex, higher visual cortex, parietal cortex, and the superior temporal sulcus (Wong et al., 2012). James and Gauthier (2003, 2004) also found that participants who verbally learned to associate artificial objects with conceptual features showed activations in non-visual areas during subsequent, perceptual judgment on these objects, including superior temporal gyrus (hearing), inferior frontal gyrus (semantics), etc.

Perceptual expertise researchers often emphasize that experts tend to automatically process their objects of expertise in a certain way (e.g., holistically, at a subordinate level of abstraction) or by recruiting certain brain areas even without explicit task instructions or requirements (e.g., Gauthier et al., 2000; Wong et al., 2009a,b). Importantly, however, this does not mean that such processes cannot be influenced or even overridden by higher-level cognitive processing^2^. On the contrary, as described above, both training studies and studies with real-world expertise demonstrate that cognitive processing (e.g., attention shaped by the current task demand, multimodal integration, and semantics) is often engaged even in tasks requiring only perceptual judgments.

Furthermore, it has been postulated that non-visual processing not only is engaged but also plays a crucial role in shaping neural selectivity for expert object categories. For example, writing training is found to be more effective than visual practice in contributing to the formation of letter selectivity in the fusiform gyrus, indicating a close interaction between motor and perceptual areas (James and Atwood, 2009; Kersey and James, 2013). Recently, Behrmann and Plaut (2013) propose that, the selective engagement of the left and right fusiform gyri for word and face processing respectively may originate from the constraint to keep the connections between visual word processing areas and language processing areas (both left-lateralized) as short as possible. Therefore, even when accounting for selectivity in visual areas, a distributed network of brain areas should be and have been considered.

## Early perceptual expertise research focuses more on the face-selective areas in order to address the “face modularity” debate, but that does not necessitate that researchers regard face-selective areas as the only brain regions important for expert object recognition

Despite the abundant research on the interaction between visual and cognitive processing in expert object recognition, why may perceptual expertise researchers be regarded as face-centric, as in Harel et al. (2013; p.4)? It has to do with the “face modularity debate” that heat up from the late 90's in the field of face perception.

The face modularity debate concerns the nature of the fusiform face area (FFA) in face processing: Is the FFA a module specialized for face recognition, or is it responsible for expert subordinate-level recognition of any objects? As stated in Bukach et al. (2006) and McGugin et al. (2012), the degree to which FFA activity is exclusive for faces lies in the center of the debate. In support of the latter view, perceptual expertise researchers have shown that acquisition of expertise with various object categories (e.g., cars, birds, “Digimon” cartoon characters, chess, and artificial objects like greebles) either leads to or is associated with increased selectivity in the FFA (Bilalić et al., 2011b; e.g., Gauthier et al., 1999, 2000; Grelotti et al., 2005; Xu, 2005). The modularity vs. expertise debate, however, is still ongoing (e.g., McGugin et al., 2012; Rezlescu et al., 2014). Therefore, a more accurate depiction of the field of expert object recognition is that, researchers (including those holding the modular and expertise views) have been focusing a lot on the FFA due to their research question concerning face modularity.

It is important to note that, although early perceptual expertise research focuses more on the face-selective areas in order to address the “face modularity” debate, that does not necessitate that researchers regard face-selective areas as the only brain regions important for expert object recognition. As an analogy, that one focuses on studying expert object recognition does not mean that one regards expert object recognition as the only important function of vision. As detailed in our first point, perceptual expertise researchers have been tackling issues other than the face modularity debate in recent years, and have expanded their investigations to different domains of perceptual expertise in a widespread network of brain areas.

## Conclusion

Perceptual expertise researchers have been actively investigating the neural changes associated with expertise both inside and outside of the visual cortex. Tackling the face modularity debate, the majority of early effort has been put into clarifying the nature of the FFA. However, much work has since been devoted to studying the role of other high-level cognitive factors, including the effects of task demand on both the development and expression of expertise, the involvement of visual and non-visual areas in expert object recognition, the effects of conceptual associations, the way non-visual processes helps determine the pattern of visual object selective activity, etc. In sum, there is no such thing as a rivalry between the so-called “perceptual view” and “interactive view” of expert object recognition, and the interaction between perceptual and cognitive processing has been well accommodated in perceptual expertise research.

### Conflict of interest statement

The authors declare that the research was conducted in the absence of any commercial or financial relationships that could be construed as a potential conflict of interest.

## Footnotes

^1^Here “perceptual expertise researchers” refer to those who investigate perceptual expertise in object recognition. To evaluate our interpretation of the position taken by perceptual expertise researchers in general, we encourage readers to refer to the papers we cited, including mainly but not limited to the works of researchers from the Perceptual Expertise Network (PEN).

^2^Take as an example the composite task frequently used to measure holistic processing in expert object recognition (e.g., Richler et al., 2008; Wong and Gauthier, 2010b; Wong et al., 2011). A typical observer would spontaneously process all parts of an object of expertise even though the task instruction requires the observer to focus on one part, leading to imperfect performance. However, the performance is mostly well above chance, indicating that one can override their natural tendency of holistic processing at least to a certain extent to fulfill task requirement.
